# Abcès encéphaliques: prise en charge, à propos d'une série de 82 cas

**DOI:** 10.11604/pamj.2014.18.110.2247

**Published:** 2014-06-04

**Authors:** Fahd Derkaoui Hassani, Nizare El Fatemi, Faycal Moufid, Mohammed YassadOudrhiri, Rachid Gana, Rachid El Maaqili, Fouad Bellakhdar

**Affiliations:** 1 Service de Neurochirurgie, Hôpital Ibn Sina, CHU Rabat, Faculté de Médecine et de Pharmacie de Rabat, Université Mohammed V – Souissi; 2 Service de Neurochirurgie de l'Hôpital Farabi, CHU Oujda, Faculté de Médecine de Oujda, Université Mohammed le premier

**Keywords:** abcès, cerveau, chirurgie, antibiothérapie, prise en charge, abscess, brain, surgery, antibiotherapy, management

## Abstract

Les auteurs proposent des options thérapeutiques dans la prise en charge des abcès encéphaliques, en fonction des paramètres cliniques et radiologiques des patients, en dégageant des facteurs pronostiques à long terme. Il s'agit d'une étude rétrospective incluant tous les abcès encéphaliques pris en charge, sur une période 13 ans, au service de Neurochirurgie de l'Hôpital Ibn Sina de Rabat. L'analyse a porté sur: le Glasgow coma scale (GCS), la présence de déficit neurologique, l'imagerie cérébrale avec contraste, et la recherche d'une porte d'entrée; le traitement a compris chirurgie et antibiothérapie, et le pronostic a été évalué à long terme. 82 cas d'abcès ont été analysés, un score de GCS inférieur à 12 a été trouvé dans 24%. La porte d'entrée a été d'origine ORL dans 45%. La trépanoponction seule a été décidée dans 34% des cas en situation d'urgence avec troubles de conscience. Elle a été suivie d'une exérèse de la coque dans 16% des cas lors de persistance ou récidive de la collection. La ponction stéréotaxiques a été réalisée dans 10% des cas lors de localisations éloquentes et l'exérèse d'emblée dans 38% des cas avec abcès collecté non menaçant. La mortalité opératoire a été de 1,22%, la morbidité de 19,30%. Les facteurs de bon pronostic à long terme ont été l’âge jeune, un GCS supérieur à 12 à l'admission, et la trépanoponction suivie de l'exérèse da la coque. L'amélioration du pronostic passe par le diagnostic précoce, un geste neurochirurgical précis et antibiothérapie adaptée, et le traitement des portes d'entrée.

## Introduction

L'abcès encéphalique est une suppuration intracrânienne réalisant une cavité néoformée. Il s'agit d'une urgence neurochirurgicale diagnostique et thérapeutique devenue rare dans les pays développés. Son pronostic a été complètement modifié du fait de la conjonction de plusieurs facteurs: la neuroimagerie moderne, la biologie et microbiologie avec découverte de nouveaux antibiotiques à large spectre et de bonne diffusion intracérébrale, et enfin le développement du plateau technique chirurgical notamment la chirurgie stéréotaxique. Les auteurs à travers ce travail proposent des options thérapeutiques dans la prise en charge des abcès encéphaliques, en fonction des paramètres cliniques et radiologiques des patients, en dégageant des facteurs pronostiques à long terme.

## Méthodes

Il s'agit d'une étude rétrospective menée au service de Neurochirurgie de l'Hôpital Avicenne du Centre Hospitalo-Universitaire de Rabat, entre 1996 et 2009. Tous les patients ont bénéficié d'un examen clinique et scannographique à l'admission. Les choix thérapeutiques variaient selon le tableau clinique à l'admission: traitement médical seul ou associé à un traitement chirurgical par ponction-trépanation ou exérèse par craniotomie. La surveillance a été assurée par l'examen clinique, les contrôles scannographiques à répétition, et un bilan inflammatoire. Cette surveillance a été maintenue pendant une durée variable permettant d'apprécier le pronostic à court terme (jusqu’à 1 mois après le traitement), moyen terme (1 an) et à long terme (au-delà de 1an après le traitement).

## Résultats

Notre série comporte 82 cas d'abcès encéphalique diagnostiqués et traités. L'incidence des abcès dans notre série est de l'ordre de 6,3 cas par an. Le sexe masculin est prédominant (sexe ratio: 3 /2), avec un âge moyen de 28 ± 14, 34 ans. La porte d'entrée est ORL dans presque la moitié des cas, et inconnue dans 23% des cas. Le délai du diagnostic est de 1 jour à 1 mois, avec une moyenne de15, 5 jours. Les signes cliniques révélateurs sont résumés dans le Tableau 1 avec comparaison à la littérature.

**Tableau 1:** Comparaison de la fréquence des signes cliniques à l'admission avec les données de la littérature. Le syndrome d'hypertension intracrânienne, le syndrome infectieux et le déficit neurologique représentent les signes cliniques les plus observés. (HTIC: Hypertension intracrânienne)

	HTIC	Fièvre	convulsion	Trouble de conscience	Déficit neurologique
Xiao[3]	15%	63%	16%	23%	45%
Menon[6]	78%	83%	17%	29%	2%
Tseng[4]	51,4%	?	13%	25,4%	57,7%
Faraji-Rad[5]	77%	14%	2%	33%	9%
Tayfun[9]	54%	57%	25%	44%	42%
Song[13]	87%	61%	27%	6%	21%
Chaoui[8]	83,33%	54,16%	29,16%	47,67%	65,27%
**Notre série**	**86,58%**	**78,05%**	**7,32%**	**24,39%**	**80,5%**

Le diagnostic d'orientation est fait grâce au scanner avec injection de produite de contraste. En effet, l'aspect typique au scanner d'abcès encéphalique est de 87,80% des cas. L'abcès est unique dans 74,39% des cas et multiple jusqu’à 4 lésions. La localisation est Sustentorielle (temporale) dans 29, 27% des cas. La taille est de 50mm en moyenne. L'imagerie par résonance magnétique en séquences de diffusion et métabolique (spectroscopie) est utilisée dans deux cas avec grande sensibilité et de spécificité. La biologie comporte un bilan inflammatoire perturbé avec VS et CRP élevées dans tous les cas réalisés en urgence (63%).

**Figure 1:**
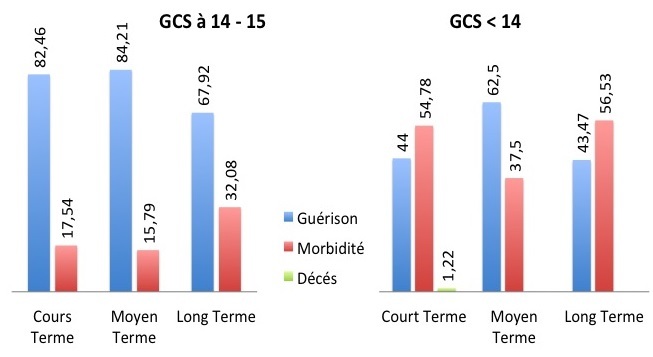
L’évolution des patiens selon le Glasgow Come Scale (GCS) permettant de comparer le pronosctic dans le court, moyen et long terme

Les différentes techniques chirurgicales employées sont résumées dans le Tableau 2. La Surveillance postopératoire est clinique (état de conscience, température, déficit), para-clinique (Scanner cérébral avec injection). Le rythme de cette surveillance est fonction de la technique chirurgicale, et de l’évolution post-opératoire immédiate. Le Pronostic est évalué à court, moyen et long termes (Figure 1).

**Tableau 2:** Comparaison de l’évolution post-opératoire des patients traités pour abcès cérébraux avec les données de la littérature

	FAVORABLE	MORBIDITE	MORTALITE
Xiao[3]	68%	13%	25%
Menon[6]	90,41%	-	9,59%
Tseng[4]	74%	16,9%	9,2%
Faraji-Rad[5]	71%	24%	5%
Tayfun[9]	57%	34%	8%
Song[13]	87,8%	7,8%	4,4%
Chaoui[8]	75%	18,3%	6,7%
**Notre série**	**60,52%**	**19,30%**	**1,22%**

## Discussion

Les abcès encéphaliques restent une pathologie cruciale en raison de sa morbidité et de sa mortalité surtout dans les pays en voie de développement (Tableau 3), Bien que rares dans les pays développés, les abcès y constituent encore un problème important [1, 2].

**Tableau 3:** Les principales séries de la littérature de prise en charge des abcès encéphaliques

Series	Année	Nombre de cas	Pays
Xiao[3]	2005	178	Taiwan
Menon[6]	2008	75	Inde
Tseng[4]	2006	142	Taiwan
Faraji-Rad[5]	2007	83	Iran
Tayfun[9]	2006	96	Turquie
Song[13]	2008	90	Chine
Chaoui[8]	2007	72	Maroc
**Notre série**	-	**82**	**Maroc**

Les abcès encéphaliques peuvent survenir à tout âge; la tranche d’âge la plus atteinte est comprise entre 11 et 20 ans et Presque 80% des cas sont observés entre la 2ème et la 4ème décennie [3–5]. Dans notre série, la tranche d’âge allant de 20 à moins de 30 ans était la plus touchée (32,02%) avec un âge moyen de 28 ans. Une prédominance masculine indépendamment à l’âge a été retrouvée dans différentes études. Les raisons d'une telle distribution restent inconnues [3, 6].

Le tableau clinique évocateur de l'abcès cérébral est la triade de BERGMAN. Le syndrome d'hypertension intracrânienne était présent dans 86,58% des cas suivi d'un déficit neurologique et de la fièvre retrouvé un peu prés chez les 4/5 des patients. 7,32% des patients se sont présentés avec la notion de survenu de crise convulsive préalable à tout traitement chirurgical. Dans la littérature, la survenue de crise convulsive est souvent plus fréquente que notre série (Tableau 1). Par contre un quart des patients ont été admis avec un trouble de conscience ce qui rejoint les chiffres retrouvés sur la littérature.

La recherche d'une porte d'entrée est systématique dans la prise en charge des patients. La porte d'entrée principale dans notre série est la sphère ORL avec 45,12% ce qui correspond à la plupart des séries décrites sur le Tableau 4. L'identification de la porte d'entrée était possible dans 76,83%. L'origine a demeurée inconnue dans le reste des cas.

**Tableau 4:** Répartition de la porte d'entrée: les infections de la sphère ORL représente la porte d'entrée la plus fréquente. L'origine inconnue vient en 2ème position dans notre série et sa fréquence s'approche de celle de plusieurs séries de la littérature

	ORL	Dentaire	Traumatisme	Méningite	chirurgie	Métastases	inconnue
Xiao[3]	14%	-	1%	-	9	26%	62%
Menon[6]	48%	1,33%	8%	-	1,33	8%	29,33%
Tseng[4]	--	-	-	-	-	27,7%	38,7%
Faraji-Rad[5]	62%	-	-	4%	18%	43%	25%
Tayfun[9]	22%	2%	15%	17%	4%	15%	25%
Song[13]	24%	-	17%	-	3%	13%	33%
Chaoui[8]	54%	4,16%	13,8%	2,77%	5,55%	2,77%	12%
**Notre série**	**45,12%**	**4,88%**	**10,97%**	**9,75%**	**2,44%**	**3,66%**	**23,17%**

Autant les signes cliniques ne sont pas spécifiques; la sémiologie morphologique en imagerie par résonance magnétique ne l'est pas aussi en matière d'abcès cérébral. L'apport de la spectroscopie et de l'imagerie de diffusion est considérable devant une lésion prenant le contraste de façon annulaire et pouvant correspondre à un abcès; une lésion secondaire, un foyer d'ischémie ou bien un site de démyélinisation [7]. En Spectroscopie; la présence d'un multiplet d'acides aminés à 0,9 ppm s'inversant avec un temps d’écho long égal à 136 ms permet d'affirmer le diagnostic. Une baisse du coefficient apparent de diffusion vient réconforter le diagnostic [7]. Uniquement 2 patients de notre série ont bénéficié d'une spectroscopie par résonnance magnétique devant la difficulté de faire cet examen dans notre contexte d'urgence. Le résultat de cet examen était d'un grand apport chez nos patients qui avaient un abcès de localisation profonde.

Une ponction seule était utilisée chez 43,91% des cas dont 34,15% des cas par stéréotaxie. Chaoui et al.[8] a utilisé cette technique chez 59,72% des cas. 15,85% des patients ont bénéficié initialement d'une ponction puis d'une exérèse devant la non amélioration Clinique et/ou radiologique du patient. Une excision chirurgicale d'emblée a été appliquée chez 37,80% des patients ce qui est plus élevé que les résultats rapportés par Hakan [9] et Chaoui[8], respectivement 16% et 11,11% des cas.

Une guérison sans séquelles est retrouvée dans 68% des cas selon la série de Maniglia [10]; 59% des cas selon la série de Donaldson [11]. La mortalité reste élevé avec 21% pour Maniglia [10] et 9,8% pour Cavusolgu H et al [12]. Dans notre série; la mortalité opératoire est de 1,22%; la morbidité est de 19,30%.

**Figure 2:**
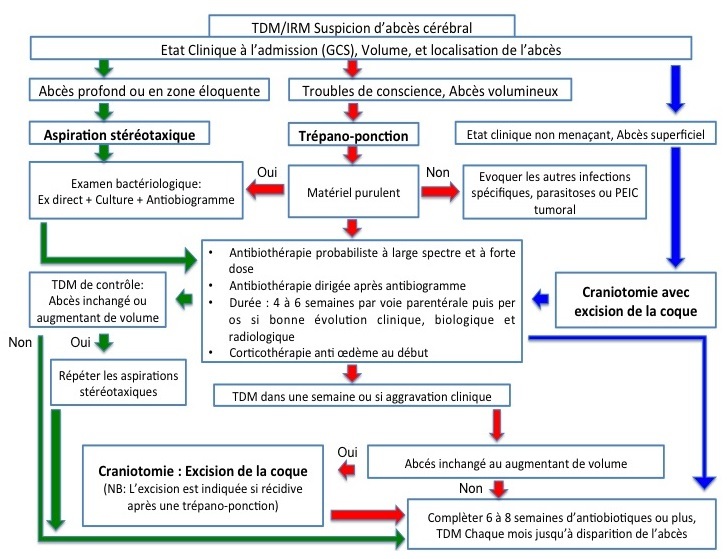
Algorithme décisionnel dans la prise en charge des abcés encéphaliques au service de neurochirurgie de l'hôpital IBN SINA de Rabat

Les facteurs pronostiques sont l’âge du patient, l’état neurologique à l'admission, et la méthode thérapeutique utilisée. Tseng [4] a conclu sur l'analyse d'une série de 144 cas d'abcès cérébraux que le sexe masculin, le GCS supérieur à 12 et l'absence d'autres complication septique. Aucune relation n'a été noté sur cette série entre le bon pronostic et l’âge, le déficit neurologique focal, les crises convulsives, les caractères de l'abcès, l'isolement de germe et la modalité thérapeutique utilisée. Tseng [4] tire l'attention que la prise en charge rapide et le contrôle efficace de l'infection sont les éléments qui peuvent assurer effectivement une bonne issue pour les patients. Xiao et al. Considère sur son papier [3] que un GCS bas, l'immunodépression et le terrain sont des facteurs de mauvais pronostic. Le Tableau 5 montre l'effet de la technique chirurgicale sur le pronostic à moyen terme. L’évolution scannographique est caractérisée par: la diminution de la taille de l'abcès (ponction); La persistance d'une image séquellaire après guérison clinique et biologique (ponction); la présence d'une cavité porencéphalique (excision); et une TDM normale. Après cette analyse comparative de notre série, un algorithme thérapeutique est proposé par les auteurs (Figure 2) [4].

**Tableau 5:** Comparaison du Pronostic à moyen terme des patients traités pour abcès cérébral selon la technique chirurgicale utilisée dans notre série

	Excision seule	Ponction et excision	Trépano-ponction seule	Ponction stéréotaxique
Guérison	**96,77%**	84,62%	82,14%	87,5%
Amélioration clinique	3, 22%	15, 38%	14,28%	12,5%
Complications	0	0	0	0
Décès	0	0	**3,6%**	0

## Conclusion

Les abcès encéphaliques sont rares mais graves, les progrès de l'imagerie ont nettement facilité le diagnostic. Le traitement associé une approche médicale et chirurgicale. Le choix du geste chirurgical dépend des caractéristiques de l'abcès et de l’état du patient. Les séquelles à long terme peuvent être lourdes justifiant le traitement curatif et préventif des portes d'entrée potentielles. De même, un diagnostic précoce de ces lésions est primordial pour l'amélioration de la morbi-mortalité d'où l'intérêt d'une sensibilisation des médecins généralistes et urgentistes au sein des différentes structures hospitalières.
